# Open‐access colonoscopy quality indicators and patient perception using split‐dose bowel preparation

**DOI:** 10.1002/jgh3.12532

**Published:** 2021-03-22

**Authors:** Nihita Manem, Katherine Donovan, David Miller, Michael Yodice, Katie Wang, Khadijat Balogun, Ghassan Kabbach, Paul Feustel, Micheal Tadros

**Affiliations:** ^1^ Albany Medical College Albany New York USA; ^2^ Division of Nephrology Stanford University School of Medicine Palo Alto California USA; ^3^ Department of Gastroenterology Eastern Connecticut Healthcare Network Manchester Connecticut USA; ^4^ Department of Internal Medicine Albany Medical Center Albany New York USA; ^5^ Department of Neuroscience and Experimental Therapeutics Albany Medical Center Hospital Albany New York USA; ^6^ Department of Gastroenterology Albany Medical Center Hospital Albany New York USA

**Keywords:** colonoscopy, colorectal cancer, patient satisfaction

## Abstract

**Background and Aim:**

Open‐access (OA) colonoscopies are defined as those scheduled without a gastrointestinal (GI) office visit. Past research has not focused on split preparation use and patient perception within OA. We aim to identify differences in bowel preparation (BP) adequacy, adenoma detection rate (ADR), self‐reported compliance, and patient perception between OA and GI providers using split prep.

**Methods:**

This was a cross‐sectional study using split BP for colonoscopies. Patients completed a survey, and demographics, BP adequacy, and ADR were recorded. BP compliance was self‐reported. Patients were asked three questions qualifying the BP instructions. Data were analyzed using chi square and Mann–Whitney tests by SPSS.

**Results:**

BP adequacy was reported for 56 of 60 patients. Twenty‐one participants (38%) were scheduled on OA, and 35 participants (62%) were scheduled after a GI office visit. Adequate BP was more frequent in 86% (18/21) of OA patients compared to 60% (21/35) in the GI group (*P* = 0.043). OA patients reported better review and explanation of the BP instructions compared to GI patients. There was no statistical difference between the demographics of the OA and GI groups or self‐reported compliance and patient understanding of instructions.

**Conclusion:**

OA scheduled colonoscopies were associated with more adequate BP. This could be explained by patients' self‐motivation or an explanation of the importance of completing BP. This study supports the use of OA procedures as a standard of care.

## Graphical abstract

We aim to identify differences in bowel preparation adequacy, adenoma detection rate, self‐reported compliance, and patient perception between open‐access (OA) and gastrointestinal (GI) providers using split preparation. OA patients reported better bowel preparation, review, and explanation of the bowel preparation instructions compared to GI patients. This study supports the use of OA procedures as a standard of care, possibly during the Coronavirus disease pandemic, which is causing a disruption in office operations.

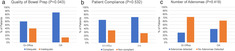

## Introduction

Open access (OA) colonoscopies are defined as those requested by a referring physician without gastroenterologist consultation or an office visit.^1^ Over the past decade, OA procedures have become more prevalent. A study conducted by Ghaoui et al. found that only one‐fifth (178/1000) of colonoscopies done in the United States are done by OA, despite it offering many advantages.^2^ This study attempts to highlight several benefits of OA colonoscopies.

The 2020 American College of Gastroenterology (ACG) guidelines state that 85% of patients with adequate bowel preparation (BP) (detection of polyps >5 mm) is acceptable.^3^, ^4^, ^5^ Increased use of OA can help achieve this target. Importantly, OA supports patient continuity under one provider and is a convenient option for primary care provider (PCP)s to stay informed about certain aspects of their patients' health (Table 1). In cases that result in unremarkable findings, the opportunity for seamless continuity of care can be an appealing option for PCPs.^6^ Moreover, medical centers are attempting to limit the number of providers exposed to patients to conserve personal protective equipment and protect from further infection spread.^7^ Patients may also want to limit the number of providers and clinic visits. In addition, studies have shown that OA procedures decrease patient costs related to possible unnecessary office consultations.^1^, ^2^, ^6^, ^8^

**Table 1:** Open access (OA) advantages and limitations

OA advantages	OA limitations
PCP continuity of care	Limited knowledge of procedures
Limits number of providers	Poor patient explanation
Decrease patient costs	Lower patient satisfaction
Eliminate unnecessary office visits	Inappropriately scheduled colonoscopies
Decreased waiting time before colonoscopy	Higher cancellation and no‐show rates
Reduced burden for gastrointestinal physicians	
Expedited screening for patients with uncomplicated histories	
Improved accessibility during Coronavirus disease 2019	

The table above describes various advantages and limitations of OA colonoscopy use.

Despite the benefits that an OA colonoscopy presents, its use has not been fully adopted.^2^ The major concern from the provider's perspective is the uncertainty of achieving a high‐quality colonoscopy.^9^ Important quality indicators that assess high‐quality colonoscopies include adequate BP and adenoma detection rate (ADR).^3^ Inadequate colonoscopy findings as a result of poor preparation may result in missed adenomas and neoplasms, possibly requiring a subsequent procedure in the near future.^5^, ^10^, ^11^ Interval colorectal cancer (CRC) is reduced with higher ADRs,^12^, ^13^ which can result from better BP.^14^ There is limited research on the quality of OA colonoscopies and the acceptance of OA referrals by gastrointestinal (GI) physicians. Split preparation has been proven to result in high‐quality colonoscopies; however, it has been found that patients seen by GI specialists are more likely to opt for split preparation compared to OA.^15^ This technique splits the bowel‐cleansing dose between the day before and day of the procedure. Split preparation reduces colon contamination with chyme from the small intestine prior to scoping due to more recent bowel cleansing.^16^ Improved compliance, ADR, and patient satisfaction has been shown with the use of split preparation.^4^, ^17^ A lack of specific knowledge of split preparation instructions from both the OA provider and patient may contribute to confusion and, ultimately, inadequate bowel preparation. Another concern regarding OA use is patient acceptance; however, more research needs to be conducted on patient self‐reported compliance and patient perception.

In this study, we attempt to fill in gaps in research. Previous studies have not addressed split preparation in terms of OA colonoscopies. We wanted to determine if a high‐quality colonoscopy was attainable with split preparation in OA. In this study, we identify differences in BP adequacy, ADR, self‐reported compliance, and patient perception between OA and GI providers.

## Materials

**Figure 1:**
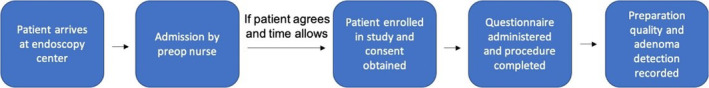
Description of patient process.

**Figure 2:**
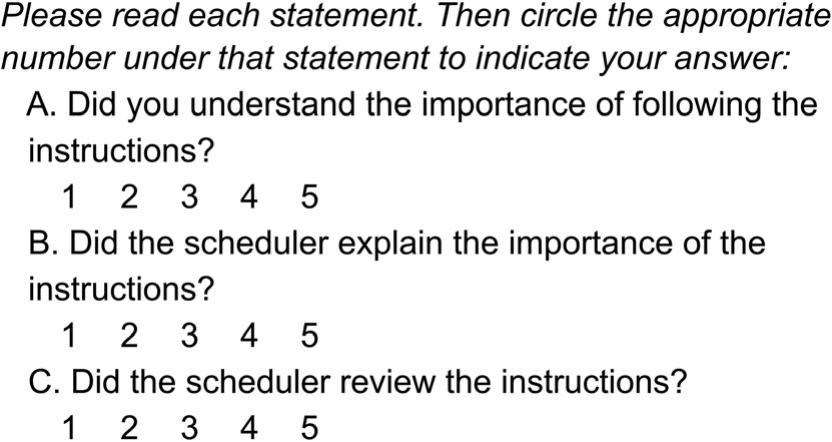
Likert scale questions assessing patient attitudes toward bowel preparation instructions.

This study was a nonrandomized comparative study of 60 patients from Albany Medical Center, a practice that performs almost 10 000 endoscopic procedures per year. All procedures performed in studies involving human participants were in accordance with the ethical standards of the institutional and/or national research committee and with the 1964 Helsinki declaration and its later amendments or comparable ethical standards. Inclusion criteria included patients who used split bowel preparation for screening and surveillance colonoscopies. If time and logistics allowed for patient participation in the study, permission was obtained by a pre‐op nurse. Informed consent was obtained from all individual participants included in the study. Surveys were administered perioperatively by a research assistant to minimize recollection or physician bias. Patient age, body mass index (BMI), gender, education level, prior c‐scope, and constipation history were collected. Patients' self‐reported compliance was recorded prior to the colonoscopy. BP adequacy and the number of adenomas detected were later retrieved by the operating physician. The operators were not notified of patient involvement in the study. Adequate BP was defined as “excellent” and “good,” and inadequate preparation was defined as “fair” or “poor.” Patients were asked three questions qualifying the BP instructions and their perception of the procedure (Fig 1)—“Does the patient understand the importance of following the instructions?,” “Did the scheduler explain the importance of the instructions?,” and “Did the scheduler review the instructions” (Fig 2). These three questions were answered by the patient using a Likert scale from one to five, with five indicating complete agreement with the statement and one indicating no agreement. SPSS 20.0 was utilized to analyze the data using chi square and Mann–Whitney tests.^18^

## Results

Complete data for BP adequacy was reported for 56 of 60 patients. The OA group included 21 participants (38%), and 35 participants (62%) were scheduled after a GI office visit. There was no noted difference in the demographic characteristics between the OA and GI office groups: age, BMI, gender, education level, history of constipation, and prior c‐scope history (*P* > 0.05) (Table 2).

**Table 2:** Demographics of patient population with chi square *P*‐values

Category	Open access	GI office	*P*‐value
Age (<60/≥60)	15/7	28/10	0.649
Body mass index (<29/≥29)	6/5	15/5	0.244
Gender (M/F)	12/9	20/16	0.907
Education level (high school/some college + graduate)	11/10	19/19	0.861
History of constipation (yes/no)	14/5	30/6	0.395
Prior c‐scope (yes/no)	12/10	24/14	0.512

The table contains the demographic information for OA and GI office patients with a *P*‐value comparing the two groups.

**Figure 3:**
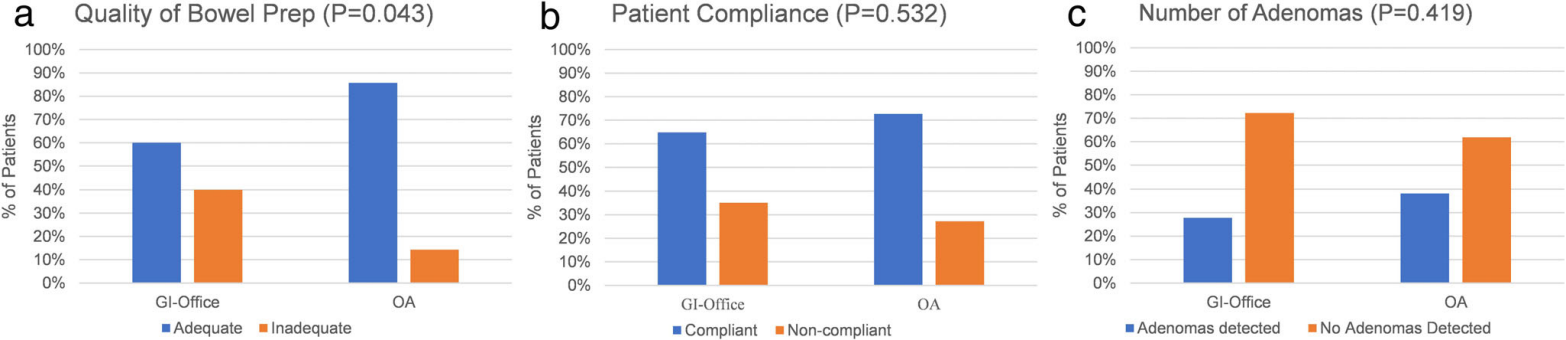
Quality of bowel preparation, patient self‐reported compliance, and number of advanced adenomas detected based on the scheduling provider.

Adequate BP was more frequent in 86% (18/21) of patients in the OA group compared to 60% (21/35) of patients in the GI office group (*P* = 0.043). Patients in the OA group had higher self‐reported compliance and adenoma detection but were not significant. Of patients in the OA group, 73% were compliant compared to 65% of patients in the GI office group (*P* = 0.532). Of patients in the OA group, 38% had adenomas detected compared to 28% of patients in the GI office group (*P* = 0.419) (Fig 3).

OA group patients reported better review and explanation of the BP instructions compared to GI office patients. The mean rank for the question “Did the scheduler review the instructions?” was 3.23 for the OA group compared to 2.04 for the GI group (*P* = 0.0008). The mean rank for “Did the scheduler explain the importance of the instructions?” was 2.82 for the OA group compared to 2.00 for the GI group (*P* = 0.0404). No statistical difference was found in how well the patients understood the importance of BP. The mean rank for understanding was 1.64 for the OA group compared to 1.42 for the GI group (*P* = 0.298) (Table 3).

**Table 3:** Mean rank and *P*‐values comparing open acess (OA) *versus* gastrointestinal (GI) office for the three Likert scale questions

	Do you understand the importance of following the instructions?		Did the scheduler review the prep instructions?		Did the scheduler explain the importance of following the instructions?	
	OA	GI	OA	GI	OA	GI
Mean rank	1.64	1.42	3.23	2.04	2.82	2.00
*P*‐value	0.298		0.0008		0.0404	

The table contains the mean rank values and *P*‐values for each likert scale question answered by the patients. Values for the OA and GI office groups are listed with a corresponding *P*‐value that compares the two groups.

## Discussion

OA was superior to GI procedures when comparing split preparation utilization. Our study focused on the use of split preparation to differentiate BP adequacy, self‐reported compliance, and ADR with regard to the referring scheduler. Given the complexity of split preparation, we were concerned that OA patients would be insufficiently prepared before colonoscopies.^15^ This study shows that OA patients successfully understood split preparation instructions compared to GI office patients, and high‐quality colonoscopies were achieved.

OA patients better perceived the explanation and review of BP protocols by their scheduler. This may have led to better BP adequacy as patients more aware of the requirements for adequate BP are more likely to take appropriate measures to ensure accurate findings. Better BP adequacy within the OA patients can also possibly be explained by patients' self‐motivation to follow directions and the desire to ensure accurate colonoscopy outcomes.

Studies have shown that busy practices have negative effects on colonoscopy outcomes.^19^ The standard for BP adequacy in clinical practice is 85%, a goal that was achieved by the OA group (86%).^4^ In contrast, low BP adequacy (60%) by GI schedulers could be a potential result of a busy practice as less time may be allotted to explaining and reviewing the requirements and process of BP.

Mean rank scores for patient understanding of the importance of BP were low. If providers are not clear about the role of BP in the colonoscopy procedure, patients may not complete the necessary steps for adequate BP. Across the board, the mean rank values for understanding, explanation, and review were lower than expected. Further research needs to be conducted to understand what factors affect patient perception and how to improve on these deficits. Specific guidelines could be implemented to help improve patient understanding.

During Coronavirus disease 2019 (COVID‐19), expanded use of OA could be of benefit to many patients. Our results, indicating better reviewing and explanation of BP instructions, shows that OA may be a suitable option in these circumstances. Several setbacks to diagnostic procedures have occurred since the recent outbreak of COVID‐19. A recommendation was made to delay nonemergent colonoscopies in March 2020, resulting in a 90% decrease in endoscopic procedures.^20^, ^21^, ^22^ As restrictions are slowly lifted, it is anticipated that endoscopy centers will be inundated with patients requiring colonoscopy.^23^ OA presents an opportunity for providers to spend more time outlining the requirements and answering any questions that patients may have surrounding BP. In addition, patients may have increased self‐motivation to avoid unnecessary office visits. Autonomous motivation has been found to play a role in increased self‐isolation during quarantine, and this could contribute to patients avoiding healthcare appointments they may deem unnecessary.^24^

Our study has several strengths. This was a cross‐sectional, unbiased study examining several effects on split BP. This is the only OA study to date that interviewed patients perioperatively to minimize recollection bias, focused on split preparation, and analyzed patient perception. Limitations to this study include the small sample size and a single‐center study population. Sixty patients provided necessary details for statistical analysis. Due to the study design, no‐show patients or cancellations were not accounted for. Self‐reported compliance may not be reliable as compliance is a subjective term. Patients can over‐ or underestimate their compliance. However, the results of this study show insight into the ability of OA schedulers to provide appropriate information that leads to adequate BP.

Our study provides potential support to the use of OA colonoscopies as COVID‐19 affects standard office and endoscopy unit procedures. During this time, it is important to highlight that OA procedures may be desirable because they increase screening while eliminating unnecessary office visits and associated costs.^1^, ^2^, ^6^, ^8^ OA patients do not require a GI consult, which decreases waiting time before scheduled colonoscopies and expedites screening and diagnostic procedures for patients with uncomplicated past medical histories.^9^ This may also help decrease delays in diagnosing GI diseases.^6^ Increasing OA use has the potential to reduce patient load and burden for GI physicians.

Following the COVID‐19 pandemic, patients may prefer to limit their exposure to medical offices and other public spaces. The results of this study may support OA colonoscopies as a method to eliminate consultations by GI providers prior to routine procedures. This could additionally alleviate scheduling difficulties in offices now restricting the number of visits and patients in an office at a given time. Another key finding in our study is illustrating that split BP can successfully be used through OA, ensuring a high‐quality colonoscopy.

While our study found benefits to OA use, past research found possible shortcomings (Fig 1). According to the 2015 American Society for Gastrointestinal Endoscopy (ASGE) guidelines, limitations to OA procedures include patient acceptance and preparedness for procedures.^25^ PCPs may have limited knowledge of the specific colonoscopy procedure, which may lead to a poor explanation of procedures and confusion. It has been found that OA patients receive less information and explanation prior to the procedure.^9^ In addition, patients seen by GI doctors were more often satisfied after colonoscopies compared to OA patients.^9^ While OA has the potential to decrease costs by eliminating unnecessary office visits, OA providers order inappropriate colonoscopies more often and, in some cases, are more prone to medical omission errors.^1^, ^26^, ^27^, ^28^, ^29^ Lack of specific experience may contribute to inappropriate referrals. These errors can lead to unnecessary exposure to the risks associated with colonoscopy, such as perforation, bleeding requiring transfusion, or serious complications like myocardial infarction (MI).^27^, ^28^, ^29^ Importantly, OA has also been shown to have higher cancellation and no‐show rates.^2^ During the COVID‐19 pandemic, it is important that practices develop effective strategies to ensure proper utilization of strained resources.

There are several recommended sites of improvement to encourage OA colonoscopy use. We propose targeting patient motivation to decrease cancellation and no‐show rates that are common among OA patients. Quality control measures should be established to avoid errors and prevent the need for subsequent procedures, ultimately reducing clinic and patient costs.^30^ Specifically, this study highlighted a need to focus on explaining and reviewing BP instructions. We suggest that more resources should be allocated to narrow gaps in the understanding of BP instructions and the colonoscopy process.

In conclusion, this study supports the use of OA procedures as a standard of care as split preparation can be effectively used in OA. Preparation adequacy may start with sufficient time spent discussing split preparation instructions with patients. It also emphasizes a need for improvements in the process of scheduling and preparing patients for their procedures. OA use can lessen the burden on the health‐care system that has occurred in response to the COVID‐19 pandemic.
